# Using Google to search for evidence: how much is enough? One center’s experience

**DOI:** 10.1186/s13643-025-02836-w

**Published:** 2025-04-22

**Authors:** Isomi M. Miake-Lye, Selene Mak, Meron M. Begashaw, Paul G. Shekelle

**Affiliations:** 1 Center for the Study of Healthcare Innovation, Implementation and Policy (CSHIIP), VA Greater Los Angeles Healthcare System, Los Angeles, CA USA; 2 Department of Health Policy and Management, UCLA Fielding School of Public Health, Los Angeles, CA USA

## Abstract

The rise of powerful search engines (e.g., Google) make the searching for gray literature more feasible within the time and resources of a typical systematic review. However, there are no hypothesis-testing studies to guide us on how to conduct such a search. It is our belief that the “best practices” for incorporating Google searches might come from the collection of experiential evidence that users have had, from which can be drawn some tentative conclusions. It is our intention with this communication to relay our experience with Google searches for five projects and the lessons we think we have learned. We invite our systematic review colleagues to contribute their own experiences and thus to build up the experiential evidence about when and how to use Google as a search engine to supplement traditional computerized database searches.

## Introduction

As the need for systematic reviews has expanded from the early focus on tightly defined pharmaceutical interventions in specific patient populations (e.g., beta-blockers use post-myocardial infarction) to more organizational or sociologic or sociotechnical interventions (quality improvement, social determinants of health interventions), the likelihood increases that relevant evidence exists outside the traditional academic or scholarly journal environment, in what is often called “the gray literature.” Whereas in the past searching for this gray literature might involve queries to potentially relevant organizations about “unpublished” studies or manual examination of conference abstracts in relevant disciplines, the rise of search engines, epitomized by Google, make the searching for gray literature more feasible. In fact, we have had peer reviewers explicitly require a Google search during revisions. As the use of Google searches is incorporated as a common systematic review search approach, the question arises — How should we do this? The current gold standard methods for searching literature databases for relevant evidence for the traditional topics of systematic reviews — biomedical interventions —were developed in a series of hypothesis-testing studies of search strategies by the Cochrane Collaboration [1–4]. But no such foundation exists for how to optimize a Google search for evidence synthesis. Thus, systematic reviewers that wish to — or are required to — incorporate a Google search for gray literature into their systematic review have no gold standard approach. Indeed, the kinds of hypothesis-testing studies done by Dr. Dickersin and colleagues in the 1990 s may not even be possible with Google searches, as the search engine is proprietary and the search’s algorithm is presumably updated at frequent intervals unknown to the user. Thus, it is our belief that the “best practices” for incorporating Google searches is not going to come from a series of hypothesis-testing studies but from the collection of experiential evidence that users have had, from which can be drawn some tentative conclusions — which in turn will need frequent updating as the search engine changes. It is our intention to relay our experience with Google searches for five separate projects and the lessons we think we have learned. We invite our systematic review colleagues to contribute their own experiences and thus to build up the experiential evidence about when and how to use Google as a search engine to supplement traditional computerized database searches.

### Some issues with google searches that make them different

Some of the issues we encountered are best understood by comparing them to the standard methods for a systematic review. In the latter, the review team must make decisions about which databases to search (normally starting with PubMed/MEDLINE and adding from there, for example, Embase, the Cochrane Database, etc.), then the construction of the search terms (for which we have the aforementioned guidance), and then how far back should the search go (to inception of the database? Or the past 10 or 20 years? etc.). Typically, one search is executed on each database, and each database searched yields hundreds or thousands of hits. All of the many thousands of hits are reviewed for relevance, and (except in rare cases) each hit is unique. In the Google search, there is no indexed database to be searched. In contrast to a traditional database search where a single, comprehensive search incorporating all the requisite terms is run, there is the possibility, even the need, to conduct multiple searches when using Google. Each search typically yields millions of hits, and clearly not all the hits can be reviewed for relevance. Lastly, not all hits are unique, in fact many of the hits keep repeating over and over in the search.

### Our experience

The five projects described here were published systematic reviews [5–9] conducted by our team as a part of the VA Evidence Synthesis Program [10] or about social determinants of health for another sponsor. As described in the original publications for each project, we used traditional evidence synthesis search approaches which primarily relied upon professional librarians developing search strategies for a set of appropriate literature databases for that specific topic. We then augmented these database searches with input from content experts. The results of these literature identification processes, as well as an abridged literature flow, can be found in Table 1.

**Table 1:** Key search characteristics and results from exemplar projects

**Project**	**Databases searched**	**Google search terms**^a^	**Citation source and disposition**			
			**Sources**	**Titles screened**	**Full text reviewed**	**Included in Review**
Panel size (5)	• PubMed • Web of Science • Scopus: Inception • Embase: Inception *Google searches conducted for manuscript only, not report*	***Primary care panel size suggested searches***^b^ • Medical Group Management Association (MGMA) panel size • Panel size primary care panel size benchmark • Patient panel size worksheet • Kaiser Permanente primary care panel size • Risk-adjusted panel size • Primary care practice size	Databases Google Other **Total**	1237 300 14 **1551**	48 20 9 **77**	24 1 3 **28**
Culture of innovation (6)	• Web of Science • Ovid MEDLINE • PsycINFO	• **Building a culture of innovation** • Seven ways to create a culture of innovation • Creating an innovation culture McKinsey • How to drive innovation culture • Culture innovation examples • How to foster innovation culture • Organizational culture and innovation • Culture strategy innovation • How to measure innovation culture	Databases Google Other **Total**	430 472 0 **902**	45 3 0 **48**	28 2 0 **30**
Food insecurity (7)	• PubMed • Cochrane • Academic Search Complete	• **Food insecurity interventions** • Evidence-based interventions for food insecurity • Evidence-based practice food insecurity • Food security interventions • Addressing food insecurity in healthcare settings	Databases Google Other **Total**	4622 250 185 **5057**	111 12 27 **150**	32 2 5 **39**
Housing insecurity (8)	• PubMed • Web of Science • EconLit	• Housing insecurity intervention health • Housing instability intervention health • Housing insecurity prevention health • Housing affordability intervention health • Housing assistance health outcomes	Databases Google Other **Total**	2294 250 0 **2544**	55 10 16 **81**	17 1 8 **26**
Loneliness (9)	• Ovid MEDLINE • Cochrane	• **Interventions for loneliness** • Interventions for loneliness and social isolation • Interventions for social isolation in the elderly • Interventions for loneliness in the elderly • Therapeutic interventions for loneliness • Strategies for reducing loneliness • How to reduce social isolation	Databases Google Other **Total**	5850 350 121 **6321**	71 4 187 **262**	25 1 34 **60**

^a^Bolded terms were the initial search terms

^b^“Primary care panel size” was used to generate the Google-recommended search terms; results from these six terms were used to screen search hits. We did not screen the hits generated with “primary care panel size.”

After traditional literature search and screening processes were underway, or in some cases complete, we conducted Google searches for these projects. The results of these can also be seen in Table 1. We conducted between 5 and 9 different searches of Google for each project, using variations on the search terms. We reviewed around 50 hits for each search, a number that was not totally arbitrary, as an earlier pilot of one Google search for one topic showed that there were no hits meeting the inclusion criteria past hit 13 [11]. Because we were aiming to identify gray literature, we did not use Google Scholar, which “provides a simple way to broadly search for scholarly literature” [12] and thus has a duplicative focus to a traditional database searches — this was borne out in our precursory explorations of the top Google Scholar hits, which were all journal articles indexed in traditional databases.

Over the course of the 5 projects, we reviewed 1622 Google search result hits, the disposition of which are displayed in a heat map (Table 2). Of these, most hits were immediately identifiable as a website, blog, or other content that did not present gray literature or peer-reviewed, published work (*n* = 1097, 68%).

**Table 2:**
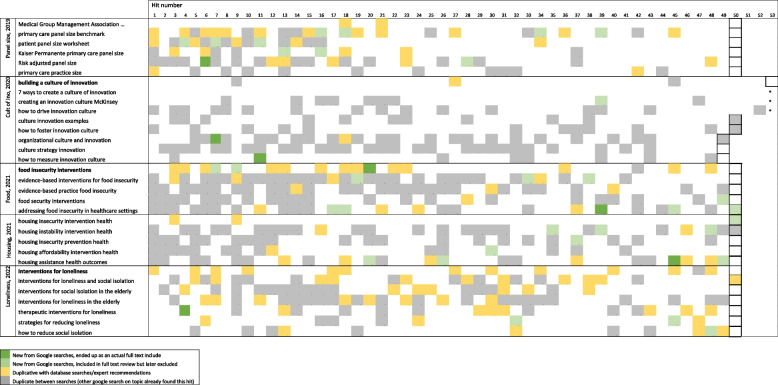
Heat map of Google search results

The two next most common types of hit were duplicative results, either between Google searches (*n* = 365, 23%) or hits that were duplicative of literature identified in our traditional literature identification process (*n* = 120, 7%). The duplicate publications were usually “secondary” articles that referenced or discussed a topic’s primary literature, which was previously found in our database search (e.g., news articles, reports, white papers, blogs). For instance, one Google search for the food insecurity project identified a narrative review published in the *Journal of Appalachian Health*. There were three subsequent results about the same review in the same Google search: the first was a link to the article on the journal’s website, the second from the University of Kentucky with a PDF version of the article, and the third was another link from the University of Kentucky with the same PDF version of the article. Thus, a very large number of the Google hits were duplicates, either of evidence already identified by our existing computerized database searches or duplicates of other hits identified earlier in the Google search process.

After excluding this content, the Google search results we incorporated into our standard screening process included 40 hits across the 5 projects (2%); 33 of them were later excluded on more detailed examination. Seven studies were included as evidence in a final review. These seven hits occurred at spots 7, 11, 14, 20, 39, 45, and 47. Of the seven hits that were fully included in our various final syntheses, five hits were identified by one search, one hit was identified by three of the six searches for the project, and another hit was identified by four of the seven searches for the project. Each review included at least one new study resulting from the Google searches, and two reviews included two new studies. A more detailed narrative of what was found for each review is available from the authors upon request. None of the included studies contributed to changing any conclusion or certainty of evidence when added to the evidence found from the traditional database searches; any changes to the synthesis were at the margin.

### Tentative lessons learned

The lessons we have learned from these Google searches are as follows:

1. Google searches for gray literature will find more eligible studies than were found in traditional computerized database searches. Each of our five examples here had at least one new study identified only through Google searching.
2. Slightly varying search questions produce somewhat different hits. This is important since all the newly included studies were identified with only one or a few variations of the search question.
3. The limit of roughly 50 hits (based on earlier pilot work) may have been too restrictive. While in three of our five examples the new studies included were identified by the 20th hit, in two of the examples, our searches identified a newly included study at hit 39 and hit 45. Until we have better evidence that can identify the kinds of topics which make it more or less likely to have early versus late relevant hits, it may be better to screen out further than 50 hits — at our center, we are going to use 100 hits as our new limit.
4. The corollary of lessons 2 and 3 is that for a given amount of team resources, we think it is better to search multiple variations of the question but limit the number of hits screened (i.e., 50 or 100). In other words, if the team devotes enough resources to screen 500 Google search hits, our experience is that it will be more productive to use 5 variations of the search question and screen 100 hits from each rather than screen 500 hits from one search.
5. Given the “black box” of Google, documentation is critical. This documentation would not be usedfor potential search replication, as a search strategy is traditionally used, but as a record of the search terms, search settings (e.g., using related search or Safe Search options), and the actual content for all hits screened. Many advanced search functions (e.g., search by document type, search within URL) are also available and could be of use for more tailored or specific searches; the use of these features, and the rationale for their use, would also be appropriate to document. Given our somewhat broad scope of gray literature, we did not employ these advanced search functions for our projects.
6. The final lesson we have learned is that Google searching for additional evidence has the same fundamental methods tension that computerized database searches have: it is always possible to search wider and deeper, and doing so will often find one or two new studies meeting inclusion criteria. But the expanded search effort is subject to the law of diminishing returns. The likelihood that new evidence which requires this amount of searching to find will change any conclusion or certainty of evidence assessment is low.

We hope that this narrative will prompt our colleagues to publish their experiences using Google as a gray literature search method, whether they have had similar or contradictory results, such that we can shortly look forward to having 100 or more case examples rather than 5 on which to support conclusions.
